# Outcome of Late Second Trimester Emergency Cerclage in Patients with Advanced Cervical Dilatation with Bulging Amniotic Membranes: A Report of Six Cases Managed at the Douala General Hospital, Cameroon

**DOI:** 10.1155/2013/843158

**Published:** 2013-11-24

**Authors:** Thomas Obinchemti Egbe, Theophile Nana Njamen, Gregory Halle Ekane, Jacques Kamgaing Tsingaing, Charlotte Nguefack Tchente, Gerard Beyiha, Esther Barla, Ernest Nyemb

**Affiliations:** ^1^Faculty of Health Sciences, University of Buea, P.O. Box 63, Buea, Cameroon; ^2^Douala General Hospital, P.O. Box 4856, Douala, Cameroon

## Abstract

*Purpose*. To show the feasibility of emergency late second trimester cerclage with advanced cervical dilatation and bulging of amniotic membranes. *Setting*. Department of Obstetrics and Gynecology of the Douala General Hospital. *Method*. This is a retrospective study of case files of patients who underwent emergency late second trimester cerclage with advanced cervical dilatation, some with bulging of fetal membranes between June 2003 and June 2010. The modified Shirodkar technique was employed in all the cases. *Results*. Altogether, six patients (100%) underwent late second trimester cervical cerclage between 24 and 26 weeks of gestational age. Four cases (66.7%) carried on their pregnancies to term that resulted in healthy live-born babies all delivered vaginally. The other two cases (33.3%) presented with preterm premature rupture of fetal membranes (PPROM) which led us to undo the stitch with eventual delivery of live-born premature fetuses which died in the neonatal intensive care unit because of complications of prematurity and neonatal infection. *Conclusion*. In experienced hands and in the absence of other risk factors like infection, the success rates of this procedure are encouraging with improved prognosis. Finally, the modified Shirodkar technique yielded excellent results in our series.

## 1. Introduction

Cervical insufficiency is a well-known cause of second trimester pregnancy loss. This is usually accompanied by painless uterine contractions effecting cervical effacement and dilatation [1]. There may also be bulging of the fetal amniotic membranes through the uterine cervix and vagina [2]. In severe cases the fetal membranes could be seen protruding through the external genitalia [3]. In such cases rupture of the fetal membranes could occur resulting in painless preterm labour and delivery of a live-born fetus [4].

However, this condition usually leads to the empiric use of tocolytic agents and cerclage. Furthermore, some studies have advocated early transvaginal sonographic follow-up with or without application of fundal pressure with the aim of detecting cases with occult cervical insufficiency, thereby indicating early cerclage. 

Cervical incompetence can occur after operations (e.g., conisation for cervical intraepithelial neoplasia and forceful cervical dilatation for advanced abortions) or as a result of congenital weakness (e.g., after intrauterine exposure to diethylstilboestrol). Uterine malformations may also give rise to preterm labour, but common ones such as a bicornuate shape are often compatible with term labour, while those such as a thin uterine septum that renders implantation insecure are rare [4]. The equivocal results of trials of cervical cerclage, however, indicate that genuine cervical insufficiency is rare, being implicated in less than 5% of cases.

Since the introduction of the cervical stitch procedure in clinical practice over 50 years ago, the efficacy of the operation has not been established by evidence-based standards for many indications. At present, five randomized clinical trials have offered significant information about elective cerclage performed for clinical indications, and the expected neonatal survival rate with properly selected elective cerclage is about 87% [5].

The 3–12% incidence of preterm labor and preterm delivery varies widely with different populations, including risk factors such as low maternal prepregnancy weight, socioeconomic status, racial and ethnic factors, maternal education, maternal work patterns, physical effort during pregnancy and especially during the third trimester, maternal sexual activity, tobacco use, interval between pregnancies, bacterial vaginosis, and other types of bacterial colonization, uterine abnormalities, number of fetuses, and more.

 Finally, other studies have not found any benefits with cerclage compared with expectant management [5]. The purpose of this report is to show the feasibility of emergency late second trimester cerclage in cases with advanced cervical dilatation and bulging of fetal membranes.

## 2. Materials and Methods

### 2.1. Setting

This is an observational study carried out at the Department of Obstetrics and Gynecology of the Douala General Hospital, Cameroon, between the periods June 2002 and June 2010. It is noteworthy that the Douala General Hospital is a tertiary care centre in Cameroon covering the Central African subregion with patients coming for treatment from neighbouring countries (Chad, Gabon, Congo Republic, Equatorial Guinea, and Central African Republic) including those coming from other towns in Cameroon.

### 2.2. Methods

Case files of patients treated during the study period were analyzed. The patients sociodemographic data were collected including age, occupation, marital status, gravidity and gestational age based on last normal menstrual period and/or ultrasound scanning [6]. Furthermore, data regarding the current pregnancy follow-up and any other medical conditions including smoking habits were recorded. The diagnosis of cervical incompetence was by physical examination because all the patients were referred to our department from other centres already with advanced cervical effacement and dilatation, some with bulging of fetal membranes. During hospitalization, blood samples were taken for full blood count, coagulation studies, HIV, syphilis, toxoplasma, and rubella serum titres. Furthermore, thick and thin film for malaria, C-reactive protein, urinalysis and culture, electrolytes, fasting blood glucose levels.

High vaginal swaps and cultures for gonococcal, chlamydiae, mycoplasma, beta-haemolytic streptococcus, and bacterial vaginosis were taken and transabdominal ultrasound scans were done for estimating gestational age, fetal weight, position, amniotic fluid volume, placenta, fetal viability, and morphologic studies. Furthermore, all our patients who underwent cerclage did not present with signs of uterine contractions, chorioamnionitis or bleeding, and preterm premature rupture of membranes (PPROM) [7].

On admission to hospital, the patients were put to bed rest preferably in the lateral recumbent position and hydration with IV fluids 2000 mL ringers lactate per day was commenced. Furthermore, they received prophylactic tocolysis with terbutaline (Bricanyl LP) 5 mg orally twice daily and betamethazone 12 mg in two doses 12 hours apart for enhancement of fetal lung maturity. The fetal wellbeing was monitored with a hand held Huntleigh Sonicaid FD2 (Fetal dopplex II) containing a 2 MHz probe until 28-week gestation when we switched to twice daily external cardiotocographic monitoring. Maternal vital signs (blood pressure, pulse, temperature, etc.) were also monitored and finally the patients were programmed for emergency late second trimester cerclage.

**Figure 1:**
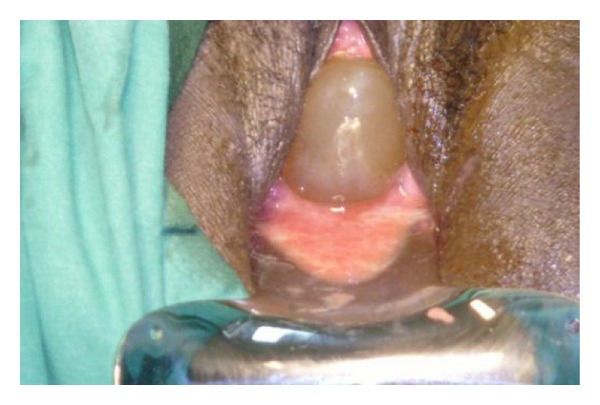
Extensive bulging of the fetal membranes.

**Figure 2:**
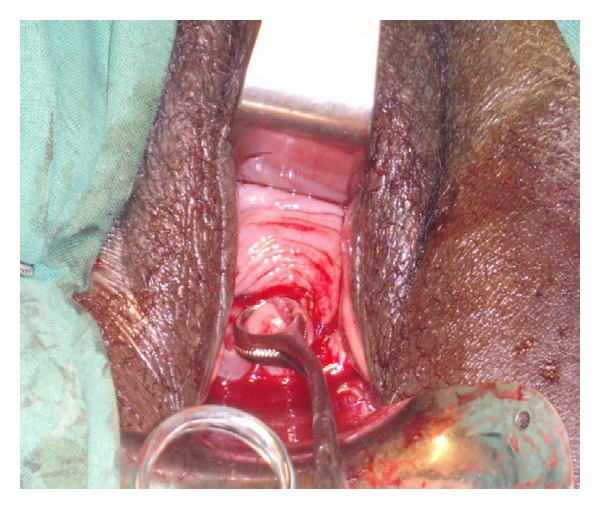
Grasping of the cervix and mobilisation of the bladder.

**Figure 3:**
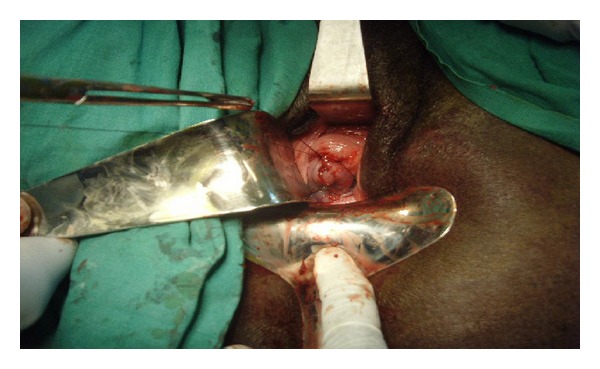
Results at the end of operation.

During the procedure patients were placed in the trendelenberg position under general anesthesia, bladder emptied, and antibiotic prophylaxis begun intraoperatively with ceftriaxone 2 gm intravenous bolus. Using vaginal retractors for exposure, the bulging membranes were pushed into the uterus using a gauze mounted to a sponge holding forceps (Figure 1). The cervix was then grasped using an atraumatic ring forceps. The modified Shirodkar procedure was used in all the cases. The bladder was mobilized cephalad (Figure 2) with submucosal placement of a Mersilene Band (Ethicon France) anterior to posterior bilaterally at the level of the internal os, tying posteriorly and burying the anterior knot at 6 o'clock (Figure 3).

Finally, all our patients were hospitalized for 48 hours after the modified Shirodkar procedure. During this period they received tocolysis (salbutamol 5 mg in 500 mL 5% Dextrose solution) and antibiotic prophylaxis with ceftriaxone 1 gm every 12 hours for 24 hours. Furthermore, they continued with tocolysis and bed rest at home for two weeks. No chronic use of tocolysis was advised. 

Follow-up was with bed rest and routine consultations every two weeks. We usually removed the cervical stitch at 36-week gestation or when indicated because of complications (rupture of membranes, chorioamnionitis, or uterine contractions). 

Ethical clearance to carry out this study was obtained from the ethical board of the Douala General Hospital, Cameroon.

## 3. Results

Altogether, six patients were analyzed during the study period and their average age was 27.6 years (range 22–35 years) while their gestational ages on admission ranged between 24 and 26 weeks. There were a total of 17 (100%) pregnancies recorded during this period. Among these pregnancies, the global number of deliveries and miscarriages in the study group between the period 2002 and 2010 was 5 (29.4%) and 12 (70%), respectively. Furthermore, most of the pregnancies 12 (70%) and abortions 8 (66.7%) were from the older (30–35 years) age group. Four deliveries were also recorded from this age group (Table 1).

 Their mean gravidity was 2.83 (17/6) pregnancies with a mean parity of 0.29 (5/17). There were no differences in the socioeconomic status of these patients. Among the six patients who underwent cerclage in our department, 4 (66.7%) carried their pregnancy to term and had normal vaginal deliveries while, in two patients (33.3%), we had to undo the stitch at 24-week, 2-day, and 26-week gestation because of preterm premature rupture of membranes (PPROM) and chorioamnionitis, respectively. These two patients were also positive for chlamydiae and *Mobiluncus species* infection, respectively, and also had bulging of fetal membranes for over 4 days prior to admission into our department (Table 1). All the other patients who took their pregnancy to term had bulging of fetal membranes into the vagina for a period no more than one day before the cerclage procedure. 

The two babies from the spontaneous abortions succumbed in the neonatal intensive care unit two and six days later, respectively. On discharge from hospital, the two patients were prescribed roxithromycin 300 mg orally per day to be taken for three consecutive weeks and metronidazole 1500 mg per day for 10 days, respectively. 

## 4. Discussion

Late second trimester cervical stitch is not a common procedure in current gynecologic practice in Cameroon. The diagnosis of cervical insufficiency is usually clinical in our country where ultrasonography is not accessible to all the regions. Cameroon being one of the low income economies, our neonatal intensive care units may not be as equipped as in the developed economies. This could explain why the two babies delivered at 24 wks 2 days and 26 weeks succumbed a few days later. On the contrary, according to literature reports from the United States, about 50% of infants born as early as 22–25 weeks of gestation may survive, and half of the survivors were without moderate to profound impairment at 18–22 months of age.

Furthermore, it has been estimated that 9.6% of all births worldwide were preterm in 2005. Approximately, 85% of this burden was concentrated in Africa and Asia, where 10.9 million births were preterm. About 0.5 million preterm births occurred in Europe and the same number in North America, while 0.9 million occurred in Latin America and the Caribbean.

 The incidence of cervical insufficiency is very difficult to determine in Cameroon because there are no studies made towards that direction and the diagnostic criteria are (facility based) not homogeneous. The limitations of this study are that the study population is too small to draw meaning conclusions of stastistical significance. Therefore, a multicentric study becomes imperative in Cameroon.

A meta-analysis of trials regarding cerclage for short cervix diagnosed by ultrasonography identified four properly conducted trials. In the total population, preterm birth at less than 35 weeks of gestation occurred in 29.2% (89/305) of the cerclage group, compared with 34.8% (105/302) of the no-cerclage groups (relative risk [RR] 0.84, 95% confidence interval [CI] 0.67–1.06). It was concluded that cerclage does not prevent preterm birth in all women with short cervical length on transvaginal ultrasonography. In the subgroup analysis of singleton gestations with short cervical length, especially those with a prior preterm birth, cerclage may reduce preterm birth, and a well-powered trial should be carried out in this group of patients. In contrast, in twins, cerclage was associated with a significantly higher incidence of preterm birth [8].

In other literature reports 225 women were studied, 152 underwent a physical examination with indicated cerclage, and 73 were managed expectantly without cerclage. Cervical dilatation, gestational age at presentation, and antenatal steroid use differed between groups. In the adjusted analyses, cerclage was associated with longer interval from presentation until delivery, improved neonatal survival, birthweight greater than 1500 g, and preterm birth less than 28 weeks, compared with expectant management. Similar results were obtained in the analyses of women with cervical dilatation between 2 and 4 cm (*n* = 122) [9].

Song et al. 2010 evaluated repeat cerclage in women with prolapsed membranes. Twenty-two women with bulging membranes after primary cerclage were offered repeat cerclage; 11 chose a repeat cerclage and 11 chose bed rest. The median gestational age at delivery, birthweight and survival rates were significantly higher in the repeat cerclage group compared to the bed rest group (mean 26.8 weeks versus 21.7 weeks, *P* = 0.04, mean birthweight 1180 g versus 491 g, *P* = 0.01 odds ratio for survival 22.0, 95% CI, 2.1–236). They concluded that early repeat cerclage under antibiotic cover may be beneficial in women with bulging membranes after a prior failing cerclage attempt [10]. In our series we had to undo the cerclage in two patients 33% because of complications leading to late second trimester abortions [11].

Other authors have reported laparoscopic abdominal cerclage with good results compared with patients put on expectant management [12]. Furthermore, there are recent reports of Da Vinci robotic assisted abdominal cerclage [13]. Nevertheless, these techniques are not well known in developing nations.

If silent membrane prolapse to or past the external os occurs at 22 weeks or before, the incidence of intrauterine bacterial colonization is 20% to 50% as reported by Romero et al. 1992 [14]. This is in conformity with our study where two patients were diagnosed to have vaginal infection with *C. trachomatis* and *Mobiluncus species*.

 The above-mentioned studies elucidate the fact that late second trimester cerclage could be beneficial in some selected cases with true cervical insufficiency. The use of prophylactic antibiotics, tocolysis, and corticosteroids for fetal lung maturity is mandatory in such cases.

## 5. Conclusion

We recommend that salvage cervical cerclage should be considered in patients with advanced cervical dilatation and bulging membranes in the second trimester. Despite overall poor prognosis in such cases successful outcomes may be obtained in selected cases especially when bulging of membranes has occurred no later than 24 hours and in the absence of maternal vaginal infection. 

We would recommend facility based introduction of ultrasound scanning for the diagnosis of the short cervix and clinical risk factors for cervical insufficiency inorder to fight against the poor morbidity/mortality related to this condition.

Follow-up of these patients after the cervical stitch with sonography could be an adjunctive modality to predict the outcomes of these pregnancies. Finally, the development of strategies for improving access to effective care in developing countries must remain a top research and operational priority. 

## Figures and Tables

**Table 1:** Maternal characteristics and outcome of pregnancies after emergency late trimester cerclage.

	Total		Maternal age (y)					
Variables			20–24		25–29		30–35	
	*N*	%	*N*.	%	*N*.	%	*N*.	%
Patients	6	100.0	2	33.3	1	16.7	3	50.0
No. of pregnancies	17	100.0	3	18.0	2	12.4	12	70.6
Total no. of deliveries	5*	100.0	1	20.0	0	0	4	80.0
No. of abortions	12	100.0	2	16.7	2	16.7	8	66.7
GA on admission (wk)								
24–25	5	100.0	2	40.0	1	20.0	2	40.0
25.1–26	1	100.0	0	0	0	0	1	100.0
Duration of bulging of membranes (day)								
≤1	4	100.0	1	25.0	0	0	3	75.0
≥4	2	100.0	1	50.0	1	50.0	0	0
Vaginal bacteriology results								
*C. trachomatis*	1	100.0	1	100.0	0	0	0	0
*Mobiluncus spp.*	1	100.0	0	0	1	100.0	0	0
Outcome of pregnancies								
Abortion	2	100.0	1	50.0	1	50.0	0	0
Vaginal delivery	4	100.0	1	25.0	0	0	3	75.0
Gestational age at delivery (wk)								
≥37	4	100.0	1	25.0	0	0	3	75.0

(i) Had one delivery before current pregnancy

(a) Global number of abortions 2002–2012 (12/17) 70.6%

(b) Global number of deliveries throughout study 2002–2010 (5/17) 29.4%

(c) Number of deliveries after modified Shirodkar cerclage (4/6) 66.7%.
